# Therapeutical Application of Caoutchouc

**Published:** 1846-06

**Authors:** 


*Therapeutical Application of Caoutchouc.*—Common salt was at one time regarded as a sovereign remedy against phthisis in all its stages; but it has resumed its old station as a culinary article, and is now beneficially employed only by cooks. Cod-liver oil succeeded the saline treatment, but this appears to be gradually losing ground, and before long will probably share the fate of common salt. A Presburg physician has, however, contrived to stumble upon a remedy which certainly has the merits of novelty and *originality* over the two above mentioned plans of treatment. This is the inward use of *caoutchouc* or Indian-rubber as a therapeutical agent! According to the account given by its discoverer, its powers are marvellous. A boy twelve years of age, labouring under hectic fever, swallowed by mistake two drachms of caoutchouc: in six weeks he was perfectly cured! A woman aged 38, who was in a very advanced stage of phthisis, having been informed of the above mentioned result, stole some pieces of caoutchouc and swallowed them: her symptoms became speedily alleviated. The caoutchouc was repeated, and she was perfectly cured!—*Gaz. Medicale.*

